# Topotecan given as a 21-day infusion in the treatment of advanced ovarian cancer

**DOI:** 10.1054/bjoc.2001.1726

**Published:** 2001-04

**Authors:** M Gore, G Rustin, J Schüller, S R Lane, S Hearn, R A Beckman, G Ross

**Affiliations:** Royal Marsden Hospital, Fulham Rd, London, SW3 6JJ, UK; Mount Vernon Hospital, Rickmansworth Rd, Northwood, Middlesex, HA6 2RN, UK; Krankenanstalt Rudolfstiftung, Juchgasse 25, Vienna, 1030, Austria; SmithKline Beecham Pharmaceuticals, 1201 South Collegeville Rd, Collegeville, PA, 19426, US; SmithKline Beecham Pharmaceuticals, New Frontiers Science Park, Third Avenue, Harlow, CM19 5AW, UK

**Keywords:** ovarian cancer, topotecan, continuous intravenous infusion

## Abstract

A phase II programme was carried out in both Europe and North America to evaluate the activity of topotecan administered as a 21-day continuous intravenous infusion to patients with recurrent ovarian cancer. The European results are reported here. Patients who had failed first line therapy with a platinum-based regimen received topotecan 0.4 mg/m^2^/day, as a 21-day infusion every 28 days. Patients were only permitted one prior regimen. 35 patients were enrolled and evaluable for response. 3 patients (8.6%) had a partial response to treatment (95% CI 1.8%, 23.1%) with a median time to response of 8.1 weeks and a median duration of response of 17.6 weeks. Response was also evaluated by CA125 and was also found to be 8%. For all 35 patients, median time to progression was 16.1 weeks and median survival was 43.6 weeks. The principal toxicity was myelosuppression although grade 4 neutropenia occurred in only 8.8% of patients (2.1% of courses) and infectious complications were relatively infrequent. Non-haematological toxicity was generally mild and mainly consisted of gastrointestinal events, alopecia and fatigue. A prolonged infusion of topotecan was well tolerated with a low incidence of severe neutropenia. Responses were seen in both North American and European patients. Response rates varied between the 2 studies possibly due to differences in patient demographics. © 2001 Cancer Research Campaign http://www.bjcancer.com


*British Journal of Cancer* (2001) **84**(8), 1043–1046
© 2001 Cancer Research Campaign
doi: 10.1054/ bjoc.2001.1726, available online at http://www.idealibrary.com on IDEAL®

### Topotecan given as a 21-day infusion in the treatment of advanced ovarian cancer

**M Gore[1], G Rustin[2], J Schüller[3], SR Lane[4], S Hearn[5], RA Beckman[4] and G Ross[5]**

[1]Royal Marsden Hospital, Fulham Rd, London, SW3 6JJ, UK; [2]Mount Vernon Hospital, Rickmansworth Rd, Northwood, Middlesex, HA6 2RN, UK; [3]Krankenanstalt Rudolfstiftung, Juchgasse 25, 1030 Vienna, Austria; [4]SmithKline Beecham Pharmaceuticals, 1201 South Collegeville Rd, Collegeville, PA 19426, US; [5]SmithKline Beecham Pharmaceuticals, New Frontiers Science Park, Third Avenue, Harlow, CM19 5AW, UK

**Summary** A phase II programme was carried out in both Europe and North America to evaluate the activity of topotecan administered as a 21-day continuous intravenous infusion to patients with recurrent ovarian cancer. The European results are reported here. Patients who had failed first line therapy with a platinum-based regimen received topotecan 0.4 mg/m$^2$/day, as a 21-day infusion every 28 days. Patients were only permitted one prior regimen. 35 patients were enrolled and evaluable for response. 3 patients (8.6%) had a partial response to treatment (95% CI 1.8%, 23.1%) with a median time to response of 8.1 weeks and a median duration of response of 17.6 weeks. Response was also evaluated by CA125 and was also found to be 8%. For all 35 patients, median time to progression was 16.1 weeks and median survival was 43.6 weeks. The principal toxicity was myelosuppression although grade 4 neutropenia occurred in only 8.8% of patients (2.1% of courses) and infectious complications were relatively infrequent. Non-haematological toxicity was generally mild and mainly consisted of gastrointestinal events, alopecia and fatigue. A prolonged infusion of topotecan was well tolerated with a low incidence of severe neutropenia. Responses were seen in both North American and European patients. Response rates varied between the 2 studies possibly due to differences in patient demographics. © 2001 Cancer Research Campaign http://www.bjcancer.com

**Keywords**: ovarian cancer; topotecan; continuous intravenous infusion

Epithelial ovarian cancer is one of the commonest gynaecological malignancies and is a leading cause of cancer death in Westernized countries. There is an absence of symptoms in its early stages and therefore, many patients present with advanced disease. Responses to first line platinum-containing chemotherapy regimens are high at 60–80% (Ozols et al, 1991; Alberts et al, 1992) however most patients ultimately relapse and 5-year survival rates are in the order of 20–30% (Ozols et al, 1992; Lorigan et al, 1996). Treatment of patients after relapse is less successful, for instance paclitaxel and topotecan offer response rates of 14–20% at the standard doses (Eisenhauer et al, 1994; ten Bokkel Huinink et al, 1997). There is a clear need for additional treatment options in these patients who have a poor prognosis.

Topotecan is a topoisomerase-1 inhibitor developed for the treatment of a range of solid tumours. In preclinical studies, its antineoplastic activity was shown to be enhanced when administered intermittently or continuously for prolonged periods. A series of phase I studies established the maximum tolerated doses (MTDs) of both daily × 5 and continuous infusion regimens. A study in pre-treated patients with solid tumours found that a 21-day continuous infusion was effective and well tolerated, and 2 of 5 patients with refractory ovarian cancers had partial responses to treatment (Hochster et al, 1994). The dose-limiting toxicity was myelosuppression and non-haematological toxicities were generally mild. In this heavily pre-treated population, the MTD of topotecan was 0.5 mg/m$^2$/day.

*Received 22 August 2000*
*Revised 19 December 2000*
*Accepted 22 January 2001*

*Correspondence to:* M Gore

A phase II programme was carried out in both Europe and North America to evaluate the activity of the 21-day infusion regimen in recurrent ovarian cancer because of the results of the phase I study. A starting daily dose of 0.4 mg/m$^2$/day was chosen due to the frequent need for dose reduction and/or interruption at the MTD dose of 0.5 mg/m$^2$/day. The results of the European part of this programme are reported here.

### PATIENTS AND METHODS

### Patients

Female patients aged at least 18 years with a histological diagnosis of metastatic epithelial ovarian cancer were entered into the study if they had failed first-line therapy with a platinum-based chemotherapy regimen, i.e. only one prior chemotherapy regimen was permitted. Patients had to have measurable disease of at least 2 cm in diameter or 1 cm in the case of skin lesions. All patients required insertion of a permanent venous access device (Hickman or medi-port) and patients of child-bearing potential were to practise adequate contraception. Written, informed consent was obtained from each patient before entry in accordance with the guidelines set out by local institutional review boards.

To be eligible for the study patients had to have an ECOG performance status ≤2, a life expectancy of at least 3 months, adequate bone marrow function (white blood cell count $\geq 3.5 \times 10^9$ l$^{-1}$, granulocytes $\geq 1.5 \times 10^9$ l$^{-1}$, platelets $\geq 100 \times 10^9$ l$^{-1}$ and haemoglobin ≥9 g dl$^{-1}$) and adequate renal and hepatic function (serum creatinine ≤130 μmol l$^{-1}$, bilirubin ≤35 μmol l$^{-1}$ and AST and ALT ≤5 × the upper limit of normal). At least 4 weeks had to have elapsed since previous chemotherapy and any previous surgery or hormonal therapy. Patients who had previous exposure to radiotherapy were not eligible. Patients with previous brain or leptomeningeal metastases or concomitant malignancies at other sites were also excluded, except for those with carcinoma in situ of the cervix, or basal or squamous cell carcinoma of the skin. The presence of severe coexisting medical problems precluded a patient from entry to the study, as did previous treatment with a camptothecin analogue.

### Treatment

Patients received a regimen of topotecan (Hycamtin™, SmithKline Beecham Pharmaceuticals) 0.4 mg/m$^2$/day given as a continuous intravenous 21-day infusion. Administration of topotecan was repeated every 28 days and treatment duration depended on the patient's response to therapy. The topotecan dose could be increased or decreased in the range 0.2–0.8 mg/m$^2$/day and/or delayed depending on toxicity assessed by the Common Toxicity Criteria. Each subsequent course of treatment began on schedule provided that there was no evidence of disease progression, the bone marrow had recovered (neutrophils >1.5 × 10$^9$ l$^{-1}$, platelets >100 × 10$^9$ l$^{-1}$, haemoglobin >9.0 g dl$^{-1}$) and there was no other clinically significant drug-related toxicity (excluding alopecia). Otherwise treatment was delayed until recovery.

A reduction in dose at the next treatment course of 0.1 mg/m$^2$/day was made in the event of grade 4 neutropenia or grade 3/4 thrombocytopenia during infusion or grade 2 neutropenia or thrombocytopenia lasting beyond day 28, or any grade 3/4 non-haematological toxicity. Similar dose reductions were made for any toxicity which delayed subsequent treatment courses. Dose escalations, in increments of 0.1 mg/m$^2$/day, were made if there was no toxicity > grade 2 for 2 consecutive courses and no dosing delays. No increases in dose were made if the patient had a complete response to treatment.

### Clinical assessments

Lesions were assessed by abdominal/pelvic CT, ultrasound or MRI scan, or chest X-ray and assessments took place either after alternate courses (CT or MRI scan) or at the end of each course (ultrasound and chest X-ray). For each patient the imaging technique was consistent throughout the study.

Full blood counts were carried out on days 1, 8, 15 and 22 of each treatment course and blood chemistry on days 1, 8 and 22. Before each course all laboratory assessments were repeated and subjective toxicity grades recorded.

### Evaluation of response

Complete response (CR) was defined as the disappearance of all measurable and evaluable disease lasting at least 4 weeks. Partial response (PR) was defined as a decrease of more than 50% in all measurable lesions for at least 4 weeks with no increase in evaluable disease or any other known lesions, or appearance of new lesions during this period. The time to response was measured from the first dose of topotecan and duration of response, both CR and PR, was measured from the time when the response was first documented to disease progression. Time to progression and survival were measured from the first administration of topotecan to progression or death due to any cause, respectively. Responses were subject to strict independent review by an external board which included a consultant radiologist.

Response rate was also evaluated by measurement of serial CA125 values. Response was defined by a 50% decrease in 2 samples, confirmed by a further sample, or a serial decrease over 3 samples of greater than 75%. The final sample had to be at least 28 days after the previous sample (Rustin et al, 2000).

### RESULTS

A total of 35 patients entered the study in Europe and demographic details are summarized in Table 1. All patients had relapsed or refractory ovarian cancer and had received first-line therapy with regimens containing carboplatin or cisplatin. Only one patient had received any other previous treatment (immunotherapy) for her relapsed disease. The majority of patients had responded to first-line chemotherapy (17% complete response, 46% partial response) and the median time to progression from first-line therapy for all patients was 26.1 weeks (range 3.7–144.7 wks). 3 patients had a platinum-free interval of >12 months.

The demographics of this population differed qualitatively from that of a similar study performed in North America (Hochster et al, 1999, personal communication) with respect to performance status, tumour bulk, and response to first line therapy. In particular, 18 of 24 (75%) North American patients either had no evidence of disease prior to first line therapy or a complete response to that initial chemotherapy. In contrast, only 6 of our 35 (17%) patients had a complete response to first line therapy ($P$ = 0.001).

### Response and survival

3 out of the 35 patients (8.6%) had a partial response to treatment (95% CI 1.8%; 23.1%) which was confirmed on independent review. 2 further claimed responses were not confirmed on

**Table 1** Patient characteristics

| | | Number (%) of patients |
|---|---|---|
| Age (years) | Mean (range) | 55 (33–74) |
| Performance status | 0 | 8 (22.9%) |
| | 1 | 26 (74.3%) |
| | 2 | 1 (2.9%) |
| Histologic grade | 1 | 2 (5.7%) |
| | 2 | 9 (25.7%) |
| | 3 | 11 (31.4%) |
| | 4 | 4 (11.4%) |
| | Unknown | 9 (25.7%) |
| Tumour size (cm) | <5 | 14 (40.0%) |
| | 5–10 | 15 (42.9%) |
| | >10 | 6 (17.1%) |
| Best response to first-line therapy | Complete response | 6 (17.1%) |
| | Partial response | 16 (45.7%) |
| | Stable disease | 9 (25.7%) |
| | Progression | 3 (8.6%) |
| | Unknown | 1 (2.9%) |
| Time to progression from end of first-line therapy | | |
| | Within 3 months | 6 (17%) |
| | 3–6 months | 9 (26%) |
| | 6–12 months | 17 (48.5%) |
| | > 12 months | 3 (8.5%) |

© 2001 Cancer Research Campaign independent review. The time to progression from the end of first-line chemotherapy in these 3 responding patients was 7 weeks, 42 weeks and 46 weeks. The time to response for these 3 patients was 7.0 weeks, 8.1 weeks and 10.9 weeks and the duration of response was 17.3 weeks, 17.6 weeks and 66.1 weeks.

25 patients were evaluable for response by CA125 and of these, 2 (8%) were responders. One of these patients responded according to both standard and CA125 criteria, the other patient responded according to CA125 but achieved only stable disease by standard criteria. This patient had a time to progression from the end of first-line chemotherapy of 36 weeks. The two remaining patients who had achieved a response by standard criteria were CA125-defined nonresponders because their CA125 levels fell only transiently.

For all patients in the study, the median time to progression was 16.1 weeks (range 0.9–74.1) and median survival was 43.6 weeks (range 2.7–95.7, Table 2).

### Toxicity

The 35 patients in this study received a total of 147 courses of topotecan (median 4 (range 1–10)). All patients were evaluable for toxicity. The principal toxicity was myelosuppression (Table 3) although grade 4 neutropenia occurred in only 8.8% of patients (2.1% of courses), all during the first course of treatment. The mean neutrophil nadir was $2.0 \times 10^9$ $l^{-1}$ (range 0.2–12.6), observed approximately 3 weeks after the start of the infusion (mean nadir day 21.5 range 6.0–47.0). Grade 3/4 infections occurred in 28.6% of patients and intravenous antibiotics were used in 12.6% of courses. However fever with infection ≥ grade 2 in association with grade 4 neutropenia occurred in only 2 courses (1.4%). These 2 patients were hospitalized and 1 died from neutropenic sepsis. 6 additional patients required admission because of infected indwelling intravenous lines.

Thrombocytopenia of grade 4 was seen in only 1 patient (2.9%) during 1 cycle (0.7%). The mean platelet nadir was $183.8 \times 10^9$ $l^{-1}$ (range 6–405) and 3 patients (8.6%) received platelet transfusions. Grade 3 anaemia occurred in 45.7% of patients (11.6% of courses) and 82.9% of patients received transfusions of red blood cells (42.2% of courses). 3 patients were hospitalized with anaemia but no grade 4 anaemia was reported.

Approximately half the courses (53.1%) were administered at the starting dose of 0.4 mg/m$^2$/day and 42.9% of courses were administered at the lower doses of 0.2 or 0.3 mg/m$^2$/day. In almost all cases the reason for dose reduction was haematological toxicity which did not appear to be cumulative. Dosing delays occurred in 42.0% of the 112 courses of therapy after course 1, approximately half of these due to haematological toxicity (22.3% of courses).

Non-haematological toxicities are shown in Table 4. Apart from alopecia, the most frequently reported toxicities were nausea (85.7% of patients), fatigue (48.5% of patients) and vomiting and abdominal pain (each in 45.7% of patients). Grade 3/4 toxicity was infrequent except for the incidence of infection (28.6%). 1 patient was withdrawn from the study due to fatigue possibly related to topotecan.

24 of the 35 patients have died, 1 from neutropenic sepsis, the remaining 23 patients due to progressive disease or other causes unrelated to study treatment.

**Table 2** Response to topotecan

| Response | All patients (*n* = 35) |
|---|---|
| Complete response (CR) | 0 |
| Partial response (PR) | 3 (8.6%) |
| Overall response | 3 (8.6%) |
| Stable disease (SD) | 9 (25.7%) |
| Time to progression (weeks): median (range) | 16.1 (0.9–74.1) |
| Survival (weeks): median (range) | 43.6 (2.7–95.7) |

**Table 3** Haematological toxicity (CTC) with grade per patient

| Toxicity | Patients (*n* = 35) | | Courses (*n* = 147)[a] | |
|---|---|---|---|---|
| | Grade 3 | Grade 4 | Grade 3 | Grade 4 |
| Neutropenia | 10 (29.4%) | 3 (8.8%) | 11 (7.6%) | 3 (2.1%) |
| Leucopenia | 7 (20.0%) | 1 (2.9%) | 9 (6.2%) | 1 (0.7%) |
| Thrombocytopenia | 5 (14.3%) | 1 (2.9%) | 6 (4.1%) | 1 (0.7%) |
| Anaemia | 16 (45.7%) | 0 (0%) | 17 (11.6%) | 0 (0%) |

[a]One or two courses for each variable with no laboratory data available.

### DISCUSSION

The activity of topotecan in recurrent ovarian cancer has already been established using a daily × 5 regimen at a topotecan dose of 1.5 mg/m$^2$/day for 5 days every 21 days (ten Bokkel Huinink et al, 1997; Bookman et al, 1998). The response rate in a phase II study with this regimen was 13.7% (Bookman et al, 1998) and 20.5% in a phase III comparative study of topotecan and paclitaxel (Gordon et al, 1998). In the latter trial this result was similar to the response rate of 14.0% obtained with paclitaxel. In this current study, topotecan 0.4 mg/m$^2$/day given as a continuous 21 day intravenous infusion every 28 days had a response rate of 8.6%. Although this is lower than the response rate achieved with a daily × 5 regimen, time to progression does not appear to have been reduced. The median time to progression of 16.1 weeks is similar to that in the previous studies of the daily × 5 regimen (12.1 and 18.9 weeks) and median survival was 43.6 weeks compared to 47 and 63 weeks (Bookman et al, 1998; Gordon et al, 1998).

Our response rate of 8.6% differs from the 35% observed in North American patients (Hochster et al, 1999). The patients in these studies differed with respect to status after first line therapy, performance status, and tumour bulk. Among these characteristics, status of CR or NED after first line therapy showed a likely correlation with response in these studies, and may help to explain the differences in response rates.

**Table 4** Non-haematological toxicity (CTC), with grade per patient

| Adverse event | Grades 1/2 | Grades 3/4 |
|---|---|---|
| Nausea | 28 (80.0%) | 2 (5.7%) |
| Alopecia | 23 (65.7%) | NA |
| Fatigue | 17 (48.6%) | 0 |
| Abdominal pain | 14 (40.0%) | 2 (5.7%) |
| Vomiting | 14 (40.0%) | 2 (5.7%) |
| Constipation | 13 (37.2%) | 2 (5.7%) |
| Infection | 5 (14.3%) | 10 (28.6%) |
| Diarrhoea | 14 (40.0%) | 0 |

NA = not applicable (grade 4 alopecia not defined).

© 2001 Cancer Research Campaign 

Topotecan administered as a 21-day continuous infusion was found to be well tolerated. The principal toxicity was myelosuppression and although infectious complications were relatively frequent this was mainly due to indwelling intravenous catheter sepsis. There was no evidence of cumulative toxicity and no G-CSF was administered either as prophylaxis or treatment. The degree of neutropenia was considerably reduced compared with the daily × 5 regimen where grade 4 neutropenia was observed in approximately 35–40% of courses (ten Bokkel Huinink et al, 1997; Bookman et al, 1998) rather than 2% as observed here. Grade 3/4 anaemia, however, was higher following the continuous infusion regimen, 46% of courses compared with approximately 10–15% for the standard regimen. Non-haematological toxicity was generally mild and mainly consisted of grade 1/2 gastrointestinal events, alopecia and fatigue.

In conclusion, a prolonged infusion of topotecan was well tolerated with a low incidence of severe neutropenia. Responses were seen in both North American and European patients but response rates varied regionally, possibly due to differences in patient demographics.

### REFERENCES

Alberts DS, Green S, Hannigan EV, O'Toole R, Stock-Novack D, Anderson P, Surwit EA, Malvlya VK, Nahhas WA and Jolles CJ (1992) Improved therapeutic index of carboplatin plus cyclophosphamide vs cisplatin plus cyclophosphamide: final report by the Southwestern Oncology Group of a phase III randomised trial in stage III and IV ovarian cancer. *J Clin Oncol* **10**: 706–717

Bookman M, Malmstrom H, Bolis G, Gordon A, Lissoni A, Krebs J and Fields S (1998) Topotecan for the treatment of advanced epithelial ovarian cancer: an open label phase II study in patients treated after prior chemotherapy that contained cisplatin or carboplatin and paclitaxel. *J Clin Oncol* **16**: 3345–3352

Eisenhauer EA, ten Bokkel Huinink WW, Swenerton KD, Gianni L, Myles J, van der Burg ME, Kerr I, Vermorken JB, Buser K and Colombo N (1994) European-Canadian randomised trial of paclitaxel in relapsed ovarian cancer: high-dose versus low-dose and long versus short infusion. *J Clin Oncol* **12**: 2654–2666

Gordon A, Carmichael J, Malfetano, Gore M, Spacynski M, Clarke-Pearson D, ten Bokkel Huinink WW, King K, Krebs J and Fields S (1998) Final analysis of a phase III, randomised study of topotecan (T) vs paclitaxel (P) in advanced epithelial ovarian carcinoma (OC): International Topotecan Study Group. *Proc Am Soc Clin Oncol* **17**: A1374

Hochster H, Liebes L, Speyer J, Sorich J, Taubes B, Oratz R, Wernz J, Chachoua A, Raphael B, Vinci R and Blum R (1994) Phase I trial of low-dose continuous topotecan infusion in patients with cancer: an active and well-tolerated regimen. *J Clin Oncol* **12**: 553–559

Hochster H, Wadler S, Runowicz C, Liebes L, Cohen H, Wallach R, Sorich J, Taubes B and Speyer J (1999) Activity and pharmacodynamics of 21 day topotecan infusion in patients with ovarian cancer previously treated with platinum-based chemotherapy. *J Clin Oncol* 1999; **17**, 8: 2553–2561

Lorigan PC, Crosby T and Coleman RE (1996) Current drug treatment guidelines for epithelial ovarian cancer. *Drugs* **51**: 571–584

Ozols RF, Hamilton TC, Hoskins WJ, Bast RC Jr and Young RC (1991) Summary of symposium: Biology and Therapy of Ovarian Cancer. *Semin Oncol* **18**: 297–306

Ozols RF, Rubin SC, Thomas GM and Robboy SJ (1997) Epithelial ovarian cancer. In: Hoskins WJ, Perez CA, Young RD (eds) *Principles and Practice of Gynecologic Oncology*, 2nd edition, pp 919–986. Lippincott: Philadelphia, PA

Rustin G, Nelstrop A, Benson S, Bond S and McClean P (2000) Selection of active drugs for ovarian cancer based on CA125 and standard response rates in phase II trials. *J Clin Oncol* **18**: 1733–1739

ten Bokkel Huinink W, Gore M, Carmichael J, Gordon A, Malfetano J, Hudson I, Broom C, Scarabelli C, Davidson N, Spaczynski M, Bolis G, Malmstrom H, Coleman R, Fields S and Heron J (1997) Topotecan vs paclitaxel for the treatment of recurrent epithelial ovarian cancer. *J Clin Oncol* **15**: 2183–2193

*© 2001 Cancer Research Campaign*

## References

[PDF_00365] Alberts D. S., Green S., Hannigan E. V., O'Toole R., Stock-Novack D., Anderson P., Surwit E. A., Malvlya V. K., Nahhas W. A., Jolles C. J. (1992). Improved therapeutic index of carboplatin plus cyclophosphamide versus cisplatin plus cyclophosphamide: final report by the Southwest Oncology Group of a phase III randomized trial in stages III and IV ovarian cancer.. J Clin Oncol.

[PDF_00371] Bookman M. A., Malmström H., Bolis G., Gordon A., Lissoni A., Krebs J. B., Fields S. Z. (1998). Topotecan for the treatment of advanced epithelial ovarian cancer: an open-label phase II study in patients treated after prior chemotherapy that contained cisplatin or carboplatin and paclitaxel.. J Clin Oncol.

[PDF_00375] Eisenhauer E. A., ten Bokkel Huinink W. W., Swenerton K. D., Gianni L., Myles J., van der Burg M. E., Kerr I., Vermorken J. B., Buser K., Colombo N. (1994). European-Canadian randomized trial of paclitaxel in relapsed ovarian cancer: high-dose versus low-dose and long versus short infusion.. J Clin Oncol.

[PDF_00385] Hochster H., Liebes L., Speyer J., Sorich J., Taubes B., Oratz R., Wernz J., Chachoua A., Raphael B., Vinci R. Z. (1994). Phase I trial of low-dose continuous topotecan infusion in patients with cancer: an active and well-tolerated regimen.. J Clin Oncol.

[PDF_00389] Hochster H., Wadler S., Runowicz C., Liebes L., Cohen H., Wallach R., Sorich J., Taubes B., Speyer J. (1999). Activity and pharmacodynamics of 21-Day topotecan infusion in patients with ovarian cancer previously treated with platinum-based chemotherapy. New York Gynecologic Oncology Group.. J Clin Oncol.

[PDF_00393] Lorigan P. C., Crosby T., Coleman R. E. (1996). Current drug treatment guidelines for epithelial ovarian cancer.. Drugs.

[PDF_00395] Ozols R. F., Hamilton T. C., Hoskins W. J., Bast R. C., Young R. C. (1991). Summary of symposium: Biology and therapy of ovarian cancer.. Semin Oncol.

[PDF_00402] Rustin G. J., Nelstrop A. E., Bentzen S. M., Bond S. J., McClean P. (2000). Selection of active drugs for ovarian cancer based on CA-125 and standard response rates in phase II trials.. J Clin Oncol.

[PDF_00406] ten Bokkel Huinink W., Gore M., Carmichael J., Gordon A., Malfetano J., Hudson I., Broom C., Scarabelli C., Davidson N., Spanczynski M. (1997). Topotecan versus paclitaxel for the treatment of recurrent epithelial ovarian cancer.. J Clin Oncol.

